# Deficiency for the Cysteine Protease Cathepsin L Impairs Myc-Induced Tumorigenesis in a Mouse Model of Pancreatic Neuroendocrine Cancer

**DOI:** 10.1371/journal.pone.0120348

**Published:** 2015-04-30

**Authors:** Nicola R. Brindle, Johanna A. Joyce, Fanya Rostker, Elizabeth R. Lawlor, Lamorna Swigart-Brown, Gerard Evan, Douglas Hanahan, Ksenya Shchors

**Affiliations:** 1 Swiss Institute for Experimental Cancer Research (ISREC), Swiss Federal Institute of Technology Lausanne (EPFL), Lausanne, Switzerland; 2 Departments of Pathology and Department of Biochemistry and Biophysics, University of California San Francisco (UCSF), San Francisco, United States of America; 3 Cancer Biology and Genetics Program, Memorial Sloan-Kettering Cancer Center, New York, New York, United States of America; University of Illinois at Chicago, UNITED STATES

## Abstract

Motivated by the recent implication of cysteine protease cathepsin L as a potential target for anti-cancer drug development, we used a conditional *MycER^TAM^;Bcl-x_L_* model of pancreatic neuroendocrine tumorigenesis (PNET) to assess the role of cathepsin L in Myc-induced tumor progression. By employing a cysteine cathepsin activity probe *in vivo* and *in vitro*, we first established that cathepsin activity increases during the initial stages of *MycER^TAM^;Bcl-x_L_* tumor development. Among the cathepsin family members investigated, only cathepsin L was predominately produced by beta-tumor cells in neoplastic pancreata and, consistent with this, *cathepsin L* mRNA expression was rapidly upregulated following Myc activation in the beta cell compartment. By contrast, cathepsins B, S and C were highly enriched in tumor-infiltrating leukocytes. Genetic deletion of cathepsin L had no discernible effect on the initiation of neoplastic growth or concordant angiogenesis. However, the tumors that developed in the cathepsin L-deficient background were markedly reduced in size relative to their typical wild-type counterparts, indicative of a role for cathepsin L in enabling expansive tumor growth. Thus, genetic blockade of cathepsin L activity is inferred to retard Myc-driven tumor growth, encouraging the potential utility of pharmacological inhibitors of cysteine cathepsins in treating late stage tumors.

## Introduction

Progression of normal cells into malignancies in humans is dependent upon acquisition of a number of functional cancer “hallmarks” including uncontrolled proliferation, suppressed cell death, increased invasion, angiogenesis, and reprogramming of biosynthetic metabolism [1]. However, the exact mechanisms by which tumors acquire these pathological attributes may be highly variable and appears to depend on both tissue of origin and the specific oncogenic mechanisms that drive each tumor.

The human cysteine cathepsins are family of 11 proteases, all of which share a conserved active site [2]. Increased expression of cysteine cathepsins (CTS) is associated with the progression of different types of human cancers (reviewed in [3, 4]). Cysteine cathepsins are well-documented mediators of lysosomal protein degradation [5] but, in addition, several cathepsins are also implicated in a number of other physiological roles, some of which involve altered subcellular localization, and secretion [3]. Among these ancillary non-lysosomal functions are MHC class II-associated antigen processing and presentation [6], skin morphogenesis [7], heart function [8], and cytotoxic T-cell-induced apoptosis [9]. In cancer, cysteine cathepsins have been identified as important contributors to tumor invasion, angiogenesis, and metastasis [4], most notably CTS B and L. A role for cathepsin B in tumor invasion and metastasis is well described [10, 11]: CTS B localizes to the invasive margin of tumors where it is produced by tumor-associated inflammatory cells [12, 13]. CTS L activity has also been demonstrated in various tumor types where it is a negative prognostic indicator in patients with breast, colorectal and head and neck cancer [14]. However, the mechanism by which cathepsin L modulates tumor progression is highly context-dependent [15] and remains controversial. While in some settings CTS L has a clear pro-tumorigenic role [12, 16], in squamous carcinomas CTS L deficiency actually promotes tumor progression [17], whereas in the *APC* ^*min*^-driven model of colon cancer loss of CTS L results in increased tumor multiplicity [18]. For example, pharmacological inhibition of cathepsin L activity is anti-angiogenic in standard *in vitro* angiogenesis assays [19] yet genetic deletion of CTS L has no effect on angiogenic switching in pancreatic neuroendocrine tumors (PNETs) [12]. Similarly, suppression of cathepsin L activity impairs invasion of glioma cells [16] but not melanoma cell lines [20]. Nonetheless, despite these disparate observations, cathepsin L is currently being evaluated as a possible target in cancer therapy and this has fostered the development of numerous cathepsin L inhibitors (review in [15]). It is therefore important to establish for which tissue type, stage of disease progression, and genetic constitution, inhibition of CTS L activity might have therapeutic potential.

The protein product of the c-*myc* gene, c-Myc, is a highly pleiotropic transcription factor that regulates expression of many, diverse genes [21]. Deregulation of the c-*myc* oncogene is implicated in driving the relentless growth of many human cancers (for comprehensive review see [22]) via its ability to drive many of the classical cancer hallmarks, including cell growth and proliferation [23, 24], angiogenesis [25, 26] and invasion [27]. Each of these hallmarks can be dissected in the synchronous *MycER* ^*TAM*^ *;Bcl-x*_*L*_ reversibly switchable model of Myc-induced beta cell tumor progression [28], a capability that has enabled us to assess the contribution made by CTS L at different stages of Myc-driven tumorigenesis. The expression of *Bcl-x*_*L*_ anti-apoptotic protein is crucial in this model for Myc-induced tumorigenesis in pancreatic islets, wherein it protects beta-cells from c-Myc-induced apoptosis [28]. Here we describe genetic studies demonstrating that blockade of CTS L activity, while inconsequential for the onset of tumor progression and exerts a profound anti-tumorigenic effect at later stages of the disease.

## Results

### Cysteine cathepsins are induced and activated in response to c-Myc activation

Cysteine cathepsin proteases, particularly CTS B and L, are frequently upregulated in various types of human cancers [29]. Moreover, in a protypical model of PNET progression—RIP1-Tag2 (RT2)—increased expression and activity of a number of cysteine cathepsins strongly correlate with advanced stages of tumorigenesis [30]. In the RT2 model, CTS B and S are produced by infiltrating immune cells and are important factors in facilitating tumor invasion. By contrast, CTS L is produced by the tumor cells and is critical for tumor maintenance [12, 13].

Deregulated activity of transcriptional factor Myc is implicated in a large number of cancers and often associated with more aggressive tumors. We sought to establish whether Myc-induced tumorigenesis in pancreatic endocrine tumors *in vivo* is associated with an increase in cysteine cathepsin L, and to determine whether, and at what stage(s) of disease progression, such CTS L might be functionally important. Activation of 4-OHT-dependent c-MycER^TAM^ in the beta-cell compartment of pancreatic islets of the *MycER* ^*TAM*^ *;Bcl-x*_*L*_ model of pancreatic neuroendocrine tumors (PNET) results in synchronous onset and progression of beta-cell proliferation, culminating in highly angiogenic and invasive tumors [26, 28]. Following systemic administration of tamoxifen (TAM), c-Myc is synchronously activated within 1–2 hours in all beta-cells, permitting facile temporal dissection of the ensuing stages of tumorigenesis *in vivo* [24].

**Fig 1:**
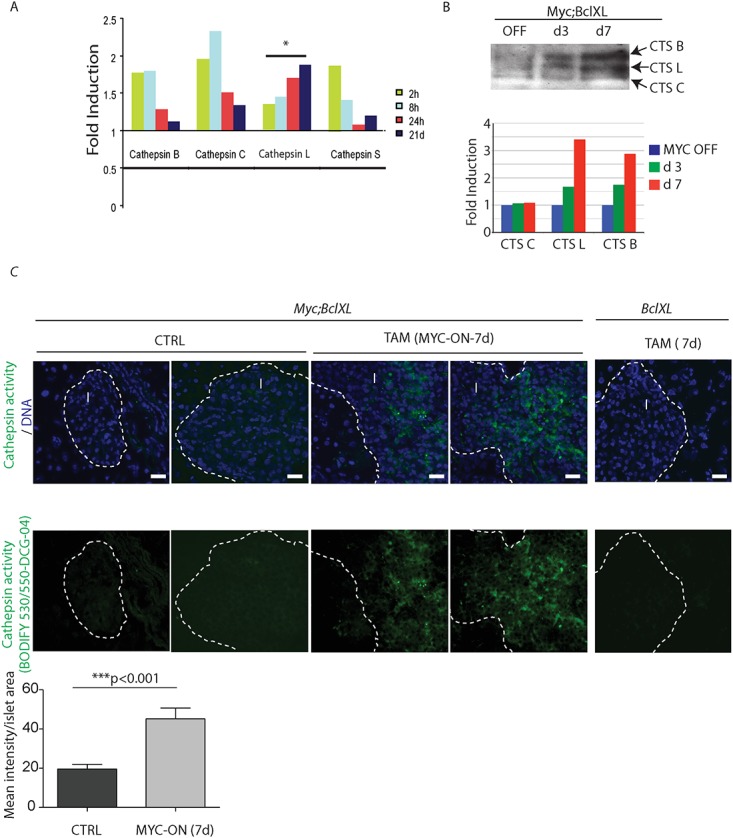
Cysteine cathepsins are rapidly induced and activated in response to c-Myc. (A) Relative fold mRNA induction of cathepsin B, C, L and S in laser-captured *MycER* ^*TAM*^ *;Bcl-xL* pancreatic islets collected at 2, 8, 24 hours and 21 days following c-Myc activation *in vivo*. Only cathepsin L mRNA is significantly and persistently upregulated following c-Myc activation. *p≤0.05 by one way ANOVA analysis. (B) Cathepsin activity profiles for the pancreatic islets from *MycER* ^*TAM*^ *;Bcl-xL* animals using DCG-04 ABP on tissue lysates. The blot shows activity in untreated islets (OFF), which increases following administration of TAM (Myc-ON) for 3 and 7 days. The activity bands corresponding to cysteine cathepsins (CTS) B, L and C are indicated. The data are representative of two independent experiments. Three animals were used for islet purifications at each data point. On the bottom, the graphical representation of cathepsin enzyme activity presented as fold of induction relative to control. The bands intensities were quantified by NIH-FIJI software. The relative expression of each cathepsin tested in the absence of Myc activation is assigned a value 1. (C) Labeling of active proteases *in vivo*. A cell-permeable fluorescent analog of DCG-04 incorporating the BODIPY fluorophore as a tag, BODIPY 530/550-DCG-04, was injected intravenously into *MycER* ^*TAM*^ *;Bcl-xL* and *Bcl-xL* mice untreated or treated with tamoxifen (TAM) for 7 days. Very little cathepsin activity was observed in islets in non-treated animals (left panel) or Myc-negative control animals injected with TAM for 7 days (right panel). However, *MycER* ^*TAM*^ *;Bcl-xL* animals had a profound increase in the cathepsin activity in the Myc-ON hyperplastic islets (middle panel, indicated by arrows). Corresponding images of pancreata counterstained with DNA-binding dye—DAPI are presented. The panels are representatives of at least three animals assayed at each data point, immunohistochemical analyses done in triplicate; seven randomized fields per analysis were considered. Scale bars are 20 μm. The islet area is indicated by the dotted line, I = islet. On the bottom, the quantification of the mean intensity of the BODIFY fluorophore in the islet area quantified by NIH-FIJI software. Statistical analysis was performed using the unpaired Student’s t test.

**Fig 2:**
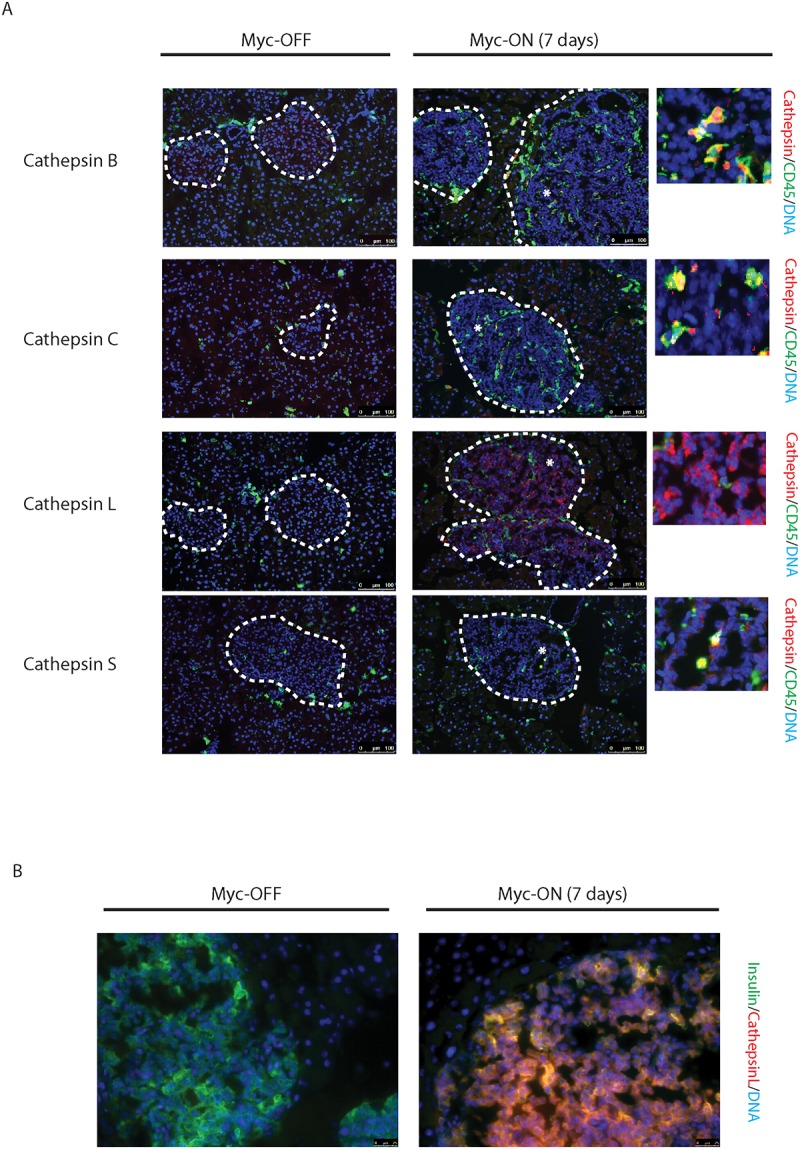
Myc induces cathepsin L expression in beta-cells of pancreatic Islets. (A) Immunohistochemical analyses for CTS B, C, L or S expression (all in red) in combination with staining for the pan-leukocyte marker CD45 (green) in pancreatic islet tumors from the *MycER* ^*TAM*^ *;Bcl-xL* animals. Pancreata were harvested from the *MycER* ^*TAM*^ *;Bcl-xL* mice treated for 7 d with TAM (Myc-On, 7 days) or control vehicle in place of TAM (Myc-OFF). The islet area is indicated by dotted line. The asterisks indicate the area of tumor represented in the insets. The panels are representatives of at least three animals assayed at each data point, all immunohistochemical analyses were done in duplicate; eight randomized fields per analysis were examined. Scale bars, 100μm. (B) Immunohistochemical analysis for cathepsin L expression in beta-cells of pancreatic islets from *MycER* ^*TAM*^ *;Bcl-xL* animals identified by insulin expression. Pancreata were collected from the animals described above. Scale bars represent 25μm. The panels are representatives of three animals assayed at each data point, all immunohistochemical analyses were done in duplicate; ten randomized fields per analysis were examined.

Analysis of a beta-cell Myc target gene database obtained from laser-captured *MycER* ^*TAM*^ *;Bcl-x*_*L*_ islets following 2, 8, 24 hours and 21 days of Myc activation [24] revealed significant and specific up-regulation of *cathepsin L* mRNA expression among the four cysteine cathepsin genes expressed in the *MycER* ^*TAM*^ *;Bcl-x*_*L*_ islets (Fig 1A). Such regulation by c-Myc is consistent with the presence of a consensus Myc-binding E-Box (*CACGTG*) element at position -458 of the *cathepsin L* promoter. We reason that the transient induction of CTS B, C and S reflects the Myc-induced recruitment of infiltrating myeloid cells [[31, 32] and Fig 2].

To determine whether induction of *CTS L* mRNA expression in the early stages of Myc-induced tumorigenesis correlates with increased cysteine cathepsin L enzymatic activity, we used DCG-04, a chemical activity-based probe (ABP) specific to the cysteine cathepsins [30, 33]. DCG-04 is based on a small molecule scaffold that binds covalently in the active sites of the cysteine cathepsins, allowing their specific identification *in vitro* and *in vivo*. Myc was activated in *MycER* ^*TAM*^ *;Bcl-x*_*L*_ mice for 3 or 7 days, corresponding to the early stages of *MycER* ^*TAM*^ *;Bcl-x*_*L*_ PNET tumor progression [26, 28]. To rule out the Myc-independent induction of CTS expression in response to intraperitoneal injection of tamoxifen, we subjected control Myc-negative *Bcl-x*_*L*_ animals to seven-day treatment with TAM (S1 Fig). The pancreata were harvested and cysteine cathepsin activity assayed with biotinylated DCG-04. Activities of both cathepsin L and B were increased following c-Myc activation *in vivo* (Fig 1B). To monitor cathepsin activity in intact animals *in vivo* we used a cell-permeable fluorescent analog of DCG-04, incorporating the BODIPY fluorophore as a tag (BODIPY 530/550-DCG-04), which was administered intravenously into *MycER* ^*TAM*^ *;Bcl-x*_*L*_ mice treatment 7 days. We detected an increase in total cystein cathepsin activity in the hyperplastic islets following Myc activation *in vivo* (Fig 1C), which was absent in TAM-treated Myc-negative control *Bcl-x*_*L*_ animals (Fig 1C). These data demonstrate that both *CTS L* mRNA expression and cysteine cathepsin activity are elevated during the early stages of Myc-induced beta cell tumourigenesis *in vivo*.

### Cysteine cathepsin L, but not cathepsins B, C or S, is produced by tumor cells

To determine the cellular source of Myc-induced cathepsin activity, Tamoxifen (TAM) was administered to *MycER* ^*TAM*^ *;Bcl-x*_*L*_ mice for 7 days and cathepsins then detected immunohistochemically in pancreas tissue sections (Fig 2A—Myc-ON). Control animals were treated with control vehicle in place of TAM (Myc-OFF). Levels of all tested cysteine cathepsins (B, C, L and S) were elevated in response to Myc activity. However, whereas CTS B, C and S expression were in the main confined to the inflammatory (CD45-positive) compartment of pancreatic tissue, cathepsin L was localized to the pancreatic islets of Langerhans. Furthermore, cathepsin L expression was limited to the insulin-producing cells in the tumor tissue, indicating a beta-cell origin of cathepsin L expression in the *MycER* ^*TAM*^ *;Bcl-x*_*L*_ tumor model (Fig 2B). Moreover, loss of CTS L activity in bone marrow derived cells in the RT2 PNET model had no impact on the number of developed tumors, tumor volume, or tumor invasion [13]. Hence, cathepsin L is expressed and activated in the cancer cells of Myc-driven PNETs *in vivo*.

### Inhibition of cysteine cathepsin L activity has no significant impact on early stages of c-Myc-induced tumorigenesis

Progression of c-Myc-induced PNET tumorigenesis is accompanied by the recruitment of a variety of inflammatory cells to the neoplastic site, which serve to support tumor growth, angiogenesis and invasion [31, 32]. The tissue microenvironments of the RT2 and the *MycER* ^*TAM*^ *;Bcl-xL* models appear to have similar properties [32], and macrophage-supplied cathepsins B and S are most likely to contribute to PNET invasion in both the *MycER* ^*TAM*^ *;Bcl-xL* and RT2 models [13]. However, tumor cell intrinsic events, including Myc-mediated regulation of cathepsin L expression, might be different between these two models, as the RT2 and the *MycER* ^*TAM*^ *;Bcl-xL* PNET cancer cells are driven by distinct oncogenes, the SV40 antigen and activated c-Myc, respectively.

**Fig 3:**
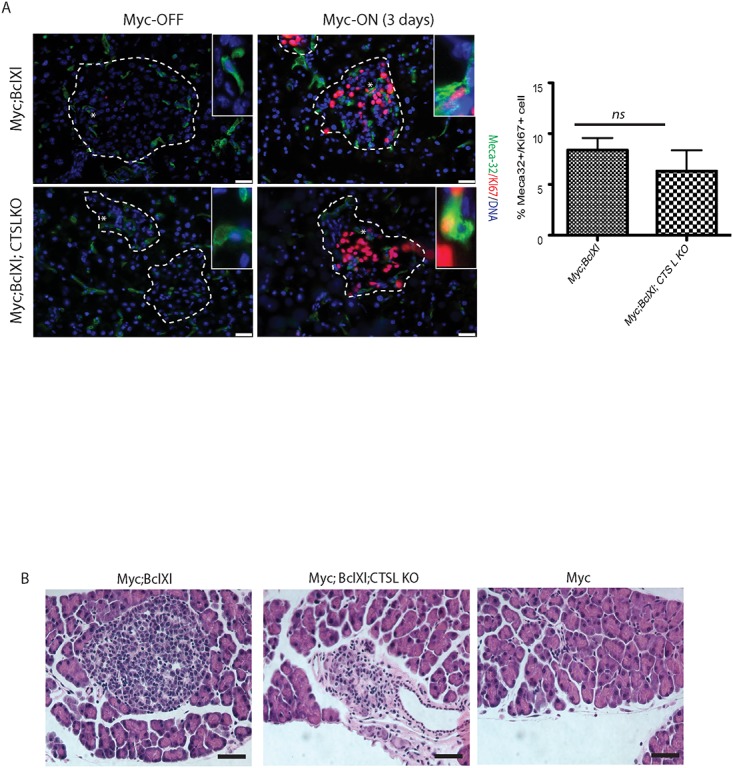
Loss of cathepsin L does not inhibit onset of Myc-induced tumorigenesis. (A) Immunohistochemical analysis of endothelial cell proliferation *in vivo*. Pancreata was isolated from the *MycER* ^*TAM*^ *;Bcl-xL* animals from CTS L WT and CTS L-deficient backgrounds untreated or treated with TAM for the duration of three days. Proliferating endothelial cells were identified by co-labeling with the endothelial marker Meca-32 (green) and Ki67 (red). The islet area is outlined by dotted lines. The asterisks indicate the magnified areas of the endothelial compartment of islets presented in the insets. The percentage of Meca-32-positive cells that also stained positive for the proliferation marker Ki67 was then determined as described in Materials and Methods section. At least three animals were assayed of each genotype all analyses done in duplicate; ten randomized fields per analysis were considered. The graph shows the mean and standard error of the mean. ns—no statistical significant difference was detected by Student’s T-test analysis. Scale bars, 25μm. (B) Representative H&E staining of pancreatic sections from *MycER* ^*TAM*^, *MycER* ^*TAM*^ *;Bcl-xL* and *MycER* ^*TAM*^ *;Bcl-xL;CTSLKO* collected from animals subjected to Myc activation for 3 days. Induction of Myc in the pancreatic islets lacking *Bcl-xL* expression (*Myc*) induces profound apoptosis and ablation of the islet beta-cell compartment as described in [28] (right panel). Loss of cathepsin L in *MycER* ^*TAM*^ *;Bcl-xL;CTSLKO* (middle panel) has no significant impact on morphology of pancreatic islets following onset of Myc-induced tumorigenesis when compared to *MycER* ^*TAM*^ *;Bcl-xL* islets (left panel). At least three animals of each genotype were assayed, eight randomized fields per analysis were considered. Scale bars, 25μm.

To determine the role of CTS L activity during *MycER* ^*TAM*^ *;Bcl-xL* PNET progression, we bred the *MycER* ^*TAM*^ *;Bcl-xL* animals into the cathepsin L-deficient background. The resulting *Myc*ER^TAM^ *;Bcl-xL;Cathepsin L KO* (*Myc*ER^TAM^ *;Bcl-xL;CTSLKO*) animals were phenotypically similar to *CTSL KO* littermates [7] in the absence of c-MycER^TAM^ activation. To assess the effect of CTS L-deficiency on the early stages of Myc-induced beta-cell tumourigenesis, *Myc;Bcl-xL;CTSLKO* and *Myc;Bcl-xL* animals were subjected to treatment with TAM for the duration of three days. This treatment induced proliferation in the beta-cell compartment of pancreatic islets accompanied by tumor angiogenesis [26], which was independent of CTS L status (Fig 3A). In addition, the percentage of proliferating endothelial cells, was unaffected by cathepsin L deficiency (Fig 3A). Moreover, *in vivo* administration of a broad-spectrum inhibitor of cysteine cathepsin activity—JPM-OEt [34]—for a 5-day duration had no impact on the Myc-mediated sequestration of extracellular-matrix-bound VEGF into the endothelial compartment of the pancreatic islets (S2A Fig), the initiating step in PNET angiogenesis [26, 35]. Our data implies that CTS L activity is not essential for the initiation of tumor angiogenesis in the Myc-driven model of PNET. One of the downstream targets of cysteine cathepsin proteolysis in the *RT2* model is the cell-cell adhesion glycoprotein E-cadherin [12]. E-cadherin is the major adhesion molecule in epithelia [36, 37]. Loss of E-cadherin, common in cancers, is a prerequisite for beta-cell invasion and metastasis in the RT2 model [38]. In the *MycER* ^*TAM*^ *;Bcl-xL* model, E-cadherin downregulation by Myc correlates with the abrupt loss of intercellular contacts within the islet, macroscopic islet expansion, and local invasion of the pancreas [28]. Immunohistochemical analysis of E-cadherin expression in the *Myc;Bcl-xL* pancreatic islets revealed rapid and sustained loss of cell surface E-cadherin staining within the pancreatic islets following three days of c-Myc activation, independent of cysteine cathepsin activity (S2B Fig), suggesting that the requirements for Myc-induced CTS L activity in the *Myc;Bcl-xL* model might be distinct from those in the RT2 model.

Several contrasting reports implicate cathepsin L as either a positive [39, 40] or a negative [16] regulator of the anti-apoptotic factor *Bcl-xL*. As noted above, expression of this anti-apoptotic protein is necessary for Myc-induced tumorigenesis in pancreatic islets, wherein it protects beta-cells from c-Myc-induced cell death ([28] and Fig 3B). Following a three-day-activation of Myc *in vivo*, the Bcl-xL protein levels remained unchanged in the *Myc*ER^TAM^ *;Bcl-xL;CTSLKO* islets when compared to the tissue collected from the *CTSLWT;MycER* ^*TAM*^ *;Bcl-xL* animals. Moreover, histopathological analysis of the pancreata collected from *MycER* ^*TAM*^ *;Bcl-xL;CTSLKO*, *MycER* ^*TAM*^ *;Bcl-xL* and *MycER* ^*TAM*^ *-only (Bcl-xL* transgene-negative) animals following three days of Myc-activation revealed that while the *Bcl-xL*-negative Myc-expressing islets rapidly involute following Myc-activation as described in [28], Myc-activation had no profound effect on the morphology of the *MycER* ^*TAM*^ *;Bcl-xL;CTSLKO* islets (S3 Fig). In summary, our data suggest that inhibition of cathepsin L activity both by genetic and pharmacological means has no significant impact on the onset of Myc-induced tumorigenesis *in vivo*.

### Loss of cathepsin L inhibits late stages of PNET progression

**Fig 4:**
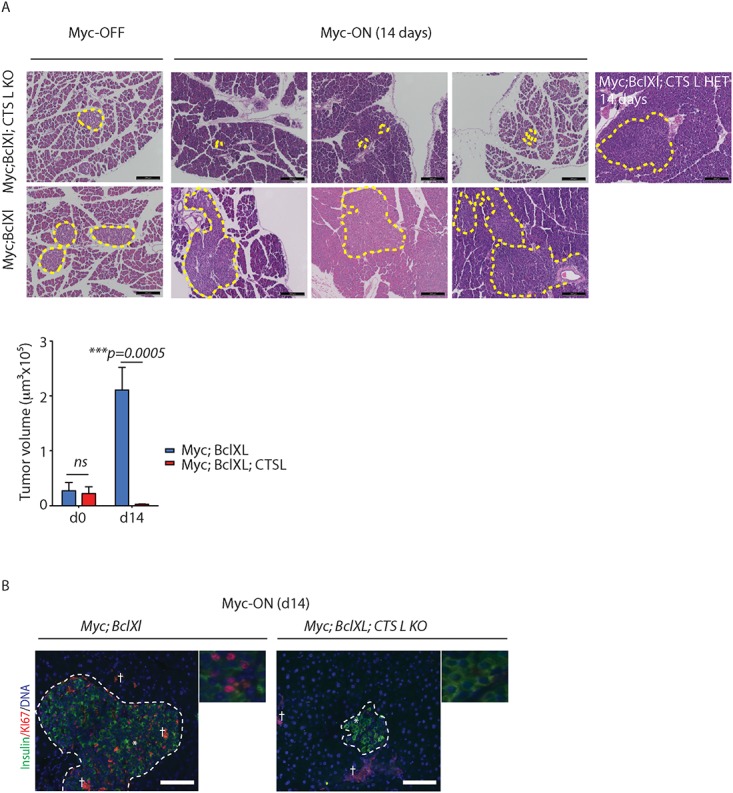
Loss of cathepsin L inhibits Myc-induced tumorigenesis in the *MycER* ^*TAM*^ *;*Bcl-xL pancreatic neuroendocrine cancer model. (A) Histopathological analysis by H&E staining of pancreatic neurodendocrine tumors from the *MycER* ^*TAM*^ *;Bcl-xL* and *MycER* ^*TAM*^ *;Bcl-xL;CTSLKO* animals untreated (Myc-OFF) or treated with TAM for 14 days (Myc-ON, 14 days). The tumor area is outlined by dotted lines. Images represent pancreata collected from three animals of each genotype. Pancreata collected from animals that retain one functional *cathepsin L* allele—*Myc;Bcl-xL;CTSL* HET (*CTSL* ^*+/-*^ *)* are presented as a control. Four animals of each genotype were assayed; at least 17 tumors for each genotype were analyzed. Scale bars, 200μm. On the bottom, graph shows quantification of pancreatic islet volume calculated using FIJI software as described in Materials and Methods section. Statistical significance was assessed using the Student T-test). (B) Immunohistochemical analysis of insulin (green) and proliferation marker KI67 (red) in pancreata collected from *MycER* ^*TAM*^ *;Bcl-xL* and *MycER* ^*TAM*^ *;Bcl-xL;CTSLKO* animals treated as described above. The tumor area is outlined by dotted lines. The asterisks indicate the enlarged areas presented. †—Non-specific staining due to tissue autofluorescence. Three animals of each genotype were assayed; all analyses done in duplicate; six randomized fields per analysis were considered. Scale bars, 100μm.

Since CTS L activity was not crucial for the initiation of Myc-driven PNET tumorigenesis, we proceeded to investigate whether sustained inactivation of CTS L modulates later stages of tumor progression. Activation of MycER^TAM^ for the duration of two weeks in the *MycER* ^*TAM*^ *;Bcl-xL* model triggers rapid and uniform progression of islets into invasive tumors [28]. We therefore subjected the *MycER* ^*TAM*^ *;Bcl-xL* mice from the cathepsin L-deficient, cathepsin L-heterozygous and cathepsin L-competent backgrounds to treatment with TAM for fourteen consecutive days (Myc-ON). While cathepsin L-deficiency had no profound effect on pancreatic tissue pathology in the absence of MycER^TAM^ activation (Fig 4A), or following three-day exposure to TAM (Fig 3B) when compared to cathepsin L wild-type (WT) counterparts, the formation of Myc-induced invasive carcinomas following two-week exposure to TAM was significantly impaired by the absence of cathepsin L (Fig 4A). Specifically, an analysis of tumor size performed as described in Materials and Methods section, established that the area of the *MycER* ^*TAM*^ *;Bcl-xL;CTSLKO* lesions noted on H&E-stained sections was 1.2% (+/-0.37%) of tumors collected from the *MycER* ^*TAM*^ *;Bcl-xL* mice. We observed no dosage-specific effect of cathepsin L deficiency, as *CTSL* heterozygous animals developed tumors indistinguishable in their histopathological features from the lesions arising from the *CTSL* WT background. Intriguingly, we detected a profound deficit in Myc-induced beta-cell proliferation in *CTSL*-deficient animals after 2-weeks of MycER^TAM^ activation (0.19% of proliferating tumor cells in the *CTSLKO* mice compared to 21.6% in the *CTSL WT* lesions) (Fig 4B) that was not apparent in the early premalignant stages of Myc-induced tumorigenesis in the *MycER* ^*TAM*^ *;Bcl-xL;CTSLKO* mice (Fig 3A).

### Loss of cathepsin L alters expression of markers of autophagy and apoptosis in Myc;Bcl-xL tumors

**Fig 5:**
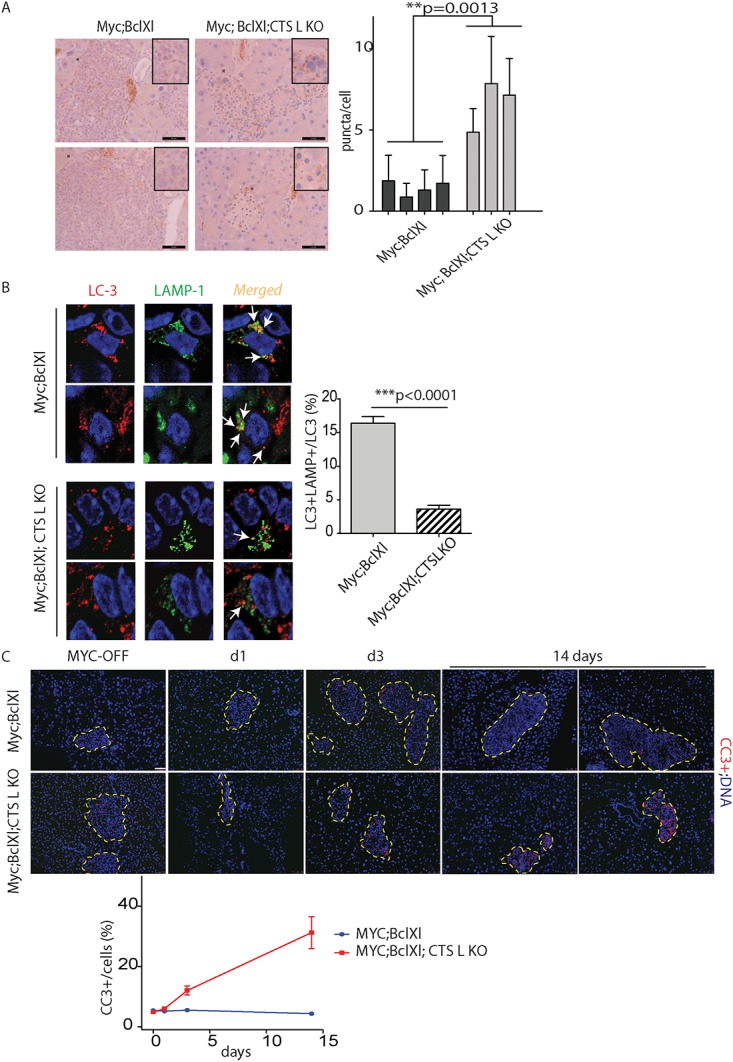
Loss of cathepsin L in Myc-induced pancreatic neuroendocrine tumors is associated with the elevated expression of markers of cell autophagy and apoptosis *in vivo*. (A) Immunohistochemical analysis of LC 5 expression in the pancreata collected from the *MycER* ^*TAM*^ *;Bcl-x*_*L*_ and *MycER* ^*TAM*^ *;Bcl-x*_*L*_ *;CTSLKO* animals untreated (Myc-OFF) or treated with TAM for the duration of 14 days (Myc-ON (14 days)). At least four animals were assayed for each genotype; immunohistochemical analyses done in duplicate; ten randomized fields per analysis were considered. The asterisks indicate areas of tumor tissue enlarged in the inset in the upper right corner. Note punctate LC3 staining in the *MycER* ^*TAM*^ *;Bcl-x*_*L*_ *;CTSLKO* animals. The graph shows quantification of LC3 puncta per cells in described tumors (n = 4 (Myc; BclXL mice) and n = 3 (Myc, Bcl Xl, CTSL mcie) at least eight independent fields for animal were considered). Statistical analysis was done by unpaired Student’s t test. Scale bars, 50 μm. (B) Immunohistochemical analysis of intracellular localization of LC-3 and the lysosomal marker LAMP1 in pancreata collected from the *MycER* ^*TAM*^ *;Bcl-x*_*L*_ and *MycER* ^*TAM*^ *;Bcl-x*_*L*_ *;CTSLKO* animals treated with TAM for the duration of 14 days determined by confocal microscopy (See Materials and Methods). White arrows marked the arrears of co-localization of LC3 and LAMP1 staining. At least three animals were assayed for each genotype; immunohistochemical analyses done in duplicate; nine randomized fields per analysis were considered. Graph shows LC3+ and LAMP1+ co-localization presented as the percentage of total LC3+ dots. *** p<0.0001 by the two-tailed Student’s t test. (C) Representative images from an IHC analysis of apoptosis assayed by activated caspase-3 (CC-3) staining in pancreata collected from the the *MycER* ^*TAM*^ *;Bcl-x*_*L*_ and *MycER* ^*TAM*^ *;Bcl-x*_*L*_ *;CTSLKO* animals treated with control vehicle (Myc-OFF) or TAM for the duration of 1, 3 or 14 consecutive days. Islet area is outlined by dotted line. At least three animals were assayed for each genotype and time point. Graph shows quantification of CC3+ cells in islet area. Scale bars = 50 μm.

Cathepsin L is a lysosomal endopeptidase that has been reported to be present inside cellular lysosomes, as well as in the lysosomal membrane [41]. Cathepsin L localization, as well as the cell type of origin, may significantly alter the range of cathepsin L substrates and their contribution to cancer progression. We hypothesized that in PNET tumors, the loss of cathepsin L activity negatively impairs fusion between the lysosomal and autophagosomal compartments of the cell, resulting in the accumulation of cellular autophagosomes. We therefore investigated whether expression of autophagic markers is altered in the CTS L-deficient tumors. The LC3 protein is a mammalian homologue of yeast factor Apg8p that is essential for autophagy [42]. The conversion of soluble LC3-I to lipid bound LC3-II is associated with the formation of autophagosomes, which can be monitored by the appearance of punctate staining in the tissue. An immunohistochemical analysis of LC3 expression in the pancreata collected from the *MycER* ^*TAM*^ *;Bcl-xL* and *MycER* ^*TAM*^ *;Bcl-xL*;*CTSL*-deficient animals subjected to 14 days of Myc-activation *in vivo* revealed a significant increase in the punctate pattern of LC3 expression in the *CTSLKO* tumors (Fig 5A). However, despite the apparent increase in the autophagosome count in CTS L KO tumors, the detected autophagosomes were not fused with cellular lysosomes. Confocal microscopy analysis of the pancreata collected following Myc activation for the duration of 14 days (Fig 5B) demonstrated that only 3.6% CTS L-deficient LC3+ vehicles versus 16.36% CTSL WT LC3+ vehicles were co-localized with lysosomes identified by LAMP-1 expression (Fig 5B). Depending on the cell of origin and oncogenic drivers, the loss of cathepsin L in tissue could negatively or positively contribute to tumor cell survival [12] [17, 18]. We proceeded to investigate the effect of CTS L loss on the survival of tumor cells in the Myc-driven PNET. The *MycER* ^*TAM*^ *;Bcl-xL* and *MycER* ^*TAM*^ *;Bcl-xL*;*CTSL*-deficient animals were subjected to treatment with TAM for the duration of 1, 3 and 14 days. The pancreata from the treated animals were collected and analyzed for the expression of activated caspase 3, a marker of apoptosis. Deletion of cathepsin L in Myc-driven PNET significantly increased tumor cell death, and was more apparent during the late stages of tumor progression (Fig 5C). In summary, our data suggests that loss of CTS L in the Myc-induced tumors results in a profound growth impairment of lesions associated with the accumulation of autophagosomes, and an increase in apoptotic cell death.

## Discussion

Efforts to determine the contributing roles of active cysteine cathepsin L to the progression of human malignancies have produced opposing results [15]. In order to evaluate cathepsin L as a potential target in cancer therapies, it will be crucial to establish its role in each type of cancer and stage of tumor progression. The current study addresses the role of cysteine cathepsin L in Myc-driven neuroendocrine tumorigenesis. Activation of Myc is proposed to be an early event in human PNET progression [43].

The elevated activity of cysteine cathepsins appears to be a common event in PNET progression, as it has been observed in both the currently presented *MycER* ^*TAM*^ *;Bcl-xL* model and previously in the *RT2* model [30]. In addition, c-Myc directly induces cysteine cathepsin activity, which highlights the importance of these enzymes in PNET tumorigenesis. Our data suggest that increased cysteine cathepsin enzyme activity in c-Myc-driven tumorigenesis is mediated by two distinct mechanisms. While some family members such as cathepsins B, C and S, are supplied to the PNET tumor site by CD45+ inflammatory cells, cathepsin L is predominantly expressed by insulin-producing cancer cells. The recruitment of inflammatory cells to the tumor site in the *MycER* ^*TAM*^ *;Bcl-xL* PNET model is directed by c-Myc-induced expression of a variety of cytokines, and is crucial for tumor invasion and maintenance of the tumor-blood supply [31, 32]. It is possible that some of these functions are mediated by lysosomal proteases that may translocate to the cell surface and are secreted outside of the cell. In the extracellular environment, cysteine cathepsins were implicated in the degradation of ECM components [3, 12, 13, 30]. However, tumor cell-supplied cathepsin L appears to function intracellularly, as suggested by this study and others [12, 18].

In contrast to the constitutive *RT2* PNET model, a conditional *MycER* ^*TAM*^ *;Bcl-xL* model enables the examination of individual stages in multi-step tumor progression. Surprisingly, none of the features associated with the early stages of c-Myc-induced tumorigenesis in PNET, such as c-Myc-induced beta-cell proliferation, initiation of tumor angiogenesis, or acute loss of E-cadherin expression, was affected by deficiency in cathepsin L activity. Interestingly, in melanoma and breast cancer xenograft models, loss of cathepsin L activity impaired tumor angiogenesis [19, 44], whereas in two independent models of PNET progression, loss of cathepsin L had no effect on the onset of tumor angiogenesis ([12] and this study). It is possible that the contributing role of cathepsin L to the regulation of angiogenesis depends on the timing, and the extent of the blood supply coverage in the particular tumor type.

The distinction between tumor initiating lesions and factors required for tumor maintenance is an important issue in tumor biology. In the constitutive model of PNET progression—*RT2*—the proliferation rate in advanced stages of tumor progression (insulinomas and invasive carcinomas) in the cathepsin L-deficient background was significantly reduced by ~90% when compared to control tumors with WT *CTS L* [12]. In the *MycER* ^*TAM*^ *;Bcl-xL* model, deficiency in cathepsin L activity also had a profound effect on late stages of disease progression, manifested by ~97% of growth inhibition and partial collapse of the developed lesions. We conclude that cathepsin L is required for tumor maintenance and progression, rather than tumor initiation, during neuroendocrine tumorigenesis involving Myc-driven proliferation.

Highly proliferative tumor cells require a constant supply of nutrients to support growth, usually achieved by increased uptake of glucose and a high glycolytic rate. C-Myc orchestrates tumor metabolic adaptation by several mechanisms, including upregulation of the glucose transporter *Glut-1*, induced over-expression of metabolic enzymes, and the use of alternative sources of energy such as glutamine [45, 46]. In addition, c-Myc is reported to regulate tumor cell autophagy both *in vitro* and *in vivo* [47, 48]. On the one hand autophagy can have a tumor suppressive effect, by targeting proteins and organelles damaged by increased reactive oxygen species associated with activated oncogenes [49, 50]. Alternatively, accumulating evidence suggests that this evolutionary conserved process can promote the growth of established malignancies by providing tumor cells with a mechanism to generate alternative sources of metabolic fuel via protein and membrane recycling [51, 52]. Thus, in cells that depend on autophagy for survival, inhibition of autophagy by chloroquine [51] or knockdown of the lysosomal protein LAMP-2 [53] leads to the accumulation of defective autophagosomes and cell death.

To our knowledge, the work presented herein is the first report demonstrating a link between the c-Myc oncogene and the lysosomal protease cathepsin L in tumor cells *in vivo*. We show that deletion of cathepsin L in Myc-driven pancreatic neoplasias results in both a reduction in autolysosomal formation and an increase in tumor cell death. Though our current data does not formally demonstrate a causal relationship between cathepsin L deletion and alteration of autophagy in Myc-driven lesions, or that the increase in tumor cell death detected in late stages of tumor progression in the *MycER* ^*TAM*^ *;Bcl-xL*;*CTSL*-deficient neoplasias is a direct consequence of altered autophagic flux *in vivo*, it is reasonable to suggest that these events are intertwined [54]. The exact sequence of events underlying cathepsin L-mediated regulation of cell autophagy and cell death in Myc-driven PNETs shall be a subject of future investigations. Nonetheless, we propose that combining inhibitors of cathepsin L with other factors targeting tumor cell autophagy may have therapeutic potential for Myc-driven malignancies at advanced stages of progression, when autophagy evidently becomes important for sustaining robust cell proliferation and tumor growth.

## Materials and Methods

### Mice, tissue sample generation, manipulation and preparation

This study was approved by the Institutional Animal Care and Use Committee (IACUC) at the University of California, San Francisco, protocol number AN076148. The transgenic mice expressing switchable MycER^TAM^ and constitutive Bcl-x_L_ in their pancreatic β cells *pIns-MycER* ^*TAM*^ *;RIP7-Bcl-x*_*L*_ (*MycER* ^*TAM*^ *;Bcl-xL*) mice have been previously described and characterized [28]. MycER^TAM^ was activated in beta-cells *in situ* by daily i.p. injection of Tamoxifen (TAM) (1mg/mouse/day) dissolved in peanut oil (Sigma). TAM is metabolized in vivo to 4-OHT and has an equivalent effect to 4-OHT when administered to *MycER* ^*TAM*^ *;Bcl-xL* mice. The cathepsin L deficient animals were previously reported [7]. *In vivo* labeling and detection of cathepsin activity with BODIPY 530/550-DCG-04 was performed as described [30]. For inhibition of cathepsin activity *in vivo*, the animals were subjected to 50mg/kg/day injection with the inhibitor JPM-OEt or control vehicle (30% DMSO/70% PBS) in conjunction with TAM injections for the duration of 5 days as previously described [30]. The animals were euthanized by exposure to CO_2_.

### Histology and immunohistochemistry

Briefly, paraffin-embedded sections (10 μm) were deparaffinized and rehydrated in a series of graded alcohols and blocked for endogenous peroxidase activity. Antigen retrieval was performed in 10 mM sodium citrate (pH 6.0). Endogenous peroxidase activity was blocked with 3% H_2_O_2_ in tap water for 5 minutes. Avidin/Biotin blocking and blocking of endogenous mouse immunoglobulins in the tissue were performed respectively by Vector Avidin/Biotin blocking kit (SP-2001) and Vector MOM basic kit (Cat. No. BMK-2202), both were used according to the manufacture’s protocols. The primary antibody used: anti-LC3 5F10 mAb (nanoTools, 1:100). Sections were incubated with ABC reagent VACTASTAIN Elite ABC Standard Kit (PK-6100) and developed with diacylbutyrate (DAB). DAB-stained slides were counterstained lightly with hematoxylin.

For the OCT-embedded tissues, 10 μm sections were fixed in 1% paraformaldehyde. The primary antibodies used: anti-mouse VEGF A (RDI-mVEGFabrP1, RDI), anti-Meca-32 (550563, BD Pharmingen), anti-Ki67 (ab16667, Abcam), anti-murine CD45 (550539, BD Pharmingen), rabbit polyclonal anti-LAMP1 (ab24170, Abcam), rabbit polyclonal anti-active-caspase 3 (AF 835, R&D), and anti-cystein cathepsins antibodies—Cathepsin B (AF965), Cathepsin S (AF1183), Cathepsin C (AF1034), and Cathepsin L (AF1515) (all from R&D Systems). All were applied in blocking buffer (2.5% BSA, 5% donkey serum) for 2–16 hours. Secondary antibodies were from Dako and Molecular Probes. The cells were counterstained with DNA-binding dye—DAPI (1-236-276-001, Roche, Mannheim, Germany).

Fluorescent images were obtained using Axiovert 100 inverted microscope (Zeiss) or Leica DM5500 microscope with cameras DFC360X (fluorescent detection) and DFC 295 (colorimetric detection). Confocal images were obtained using a LSM 700 (Zeiss). Immunohistochemical analysis for each antigen was performed at least in duplicate. The quantitative analyses of immmunostained sections were performed automatically by Fuji NIH software, with algorithms provided by the Bioimaging and Optics platform at EPFL.

Endothelial cell proliferation was quantified in tissue sections by counting randomized fields of endothelial cells (Meca32+) and calculating the percentage of Ki67+ cells. At least three animals were assayed of each genotype all analyses done in duplicate; ten randomized fields per analysis were considered. Statistical significance was assessed using the Student T-test.

Analysis of size was performed with the Fiji Software package (http://rsb.info.nih.gov/ij) as follows. Ten serial H&E-stained sections of the entire pancreata from the tumor-bearing animals were scanned with a Leica DM5500 at 20x magnification. The tumors were manually outlined with Freehand Selection tool. The software then calculates the outlined area. At least 17 tumors for each genotype were analyzed. Each collected tumor was treated as an independent event. Analysis of BODIFY fluorophore mean intensity was performed with the Fiji Software package. The tumors were manually outlined with Freehand Selection tool. The software then calculates the mean intensity of the selected area. At least eight tumors for each treatment were analyzed.

### Analysis of cathepsin activity in vitro

For *in vitro* analysis, the islets collected as described [26] were homogenized on ice in lysis buffer (50mM acetate buffer [pH 5.5], 5mM DTT, 5mM MgCl_2_, 0.1% Triton X-100). Protein concentration was established by the Bradford assay. The equal amount of protein lysates were labeled with biotinylated DCG-04 ABP [30] equally loaded, and analyzed by gel electrophoresis as previously described [33].

### Laser-capture microdissection, Affymetrix GeneChip Arrays, data analysis and promoter analysis

These procedures were described in [24].

### Statistical analysis and quantification of immune-stainings

Data were presented as the means ± standard error of the mean (SEM). The statistical analysis was calculated using GraphPad Prism5 software package. The quantification of tumor proliferation and apoptosis was performed by the Fiji Software package (*http*:*//rsb*.*info*.*nih*.*gov/ij*) according to algoriphm defined by Bioimaging & Optics platform (PT-*BIOP*) at EPFL.

## Supporting Information

S1 Fig Increased cathepsin activity detected following tamoxifen-treatment *in vivo* is restricted to the *MycER* ^*TAM*^-expressing islets. Cathepsin activity profiles for the pancreatic islets from *MycER* ^*TAM*^ *;Bcl-xL* and control *Bcl-xL* background animals using DCG-04 ABP on tissue lysates. The blot shows activity in untreated islets (OFF), which increases following administration of 4-OHT (Myc-ON) at the timepoints indicated. Two independent islet purifications are presented for *MycER* ^*TAM*^ *;Bcl-xL* animals treated for 7 days. The activity bands corresponding to cysteine cathepsins (CTS) L, B and C are indicated. Molecular weight marker is indicated on the side panel. Note the lack of cathepsin L and cathepsin B activation in samples collected from control *Bcl-xL* background animals.
(TIF)

S2 Fig Inhibition of Cathepsin activity *in vivo*. (A) Inhibition of cathepsin activity does not interfere with Myc-induced redistribution of VEGF-A in islets. *MycER* ^*TAM*^ *;Bcl-x*_*L*_ animals were either untreated or treated daily with TAM for 5 days in conjunction with control vehicle (DMSO) or the broad spectrum cathepsin inhibitor JPM-OEt (JMP). At the times indicated, animals were sacrificed and pancreata isolated, sectioned and stained with anti-VEGF-A antibody (red) and Meca-32 (green). Images collected from two independent animals are presented. The panels are representatives of at least three animals assayed at each data point, immunohistochemical analyses done in duplicate; seven randomized fields per analysis were considered. Scale bars represent 100 μm. (B) Immunohistochemical analysis of E-cadherin in the pancreata collected from the animals described above. The areas positive for E-cadherin expression in inhibitor-treated mice are indicated by arrows. I-islet area is outlined by dotted line. The panels are representatives of at least three animals assayed at each data point, immunohistochemical analyses done in duplicate; five randomized fields per analysis were considered. Scale bars represent 20 μm.
(TIF)

S3 Fig Loss of cathepsin L does not affect the levels of Bcl-x_L_ expression in *MycER* ^*TAM*^ *;*Bcl-x_*L*_ islets. Immunohistochemical analysis of Bcl-xL expression in pancreatic tissues collected from *MycER* ^*TAM*^ *;Bcl-x*_*L*_ and *MycER* ^*TAM*^ *;Bcl-x*_*L*_ *;CTSLKO* animals subjected to 3-day-treatment with TAM. (Myc-ON, d3). Tissues collected from *MycER* ^*TAM*^ *;CTSLWT* islets were used as a negative control for the staining. Three animals were assayed of each genotype; seven randomized fields per analysis were considered. Scale bars represent 50 μm.
(TIF)
