# Supplementary figures and images for: Deficiency for the Cysteine Protease Cathepsin L Impairs Myc-Induced Tumorigenesis in a Mouse Model of Pancreatic Neuroendocrine Cancer
Source: PLoS One. 2015 Apr 30;10(4):e0120348. doi: 10.1371/journal.pone.0120348 (PMC4415914; doi:10.1371/journal.pone.0120348)

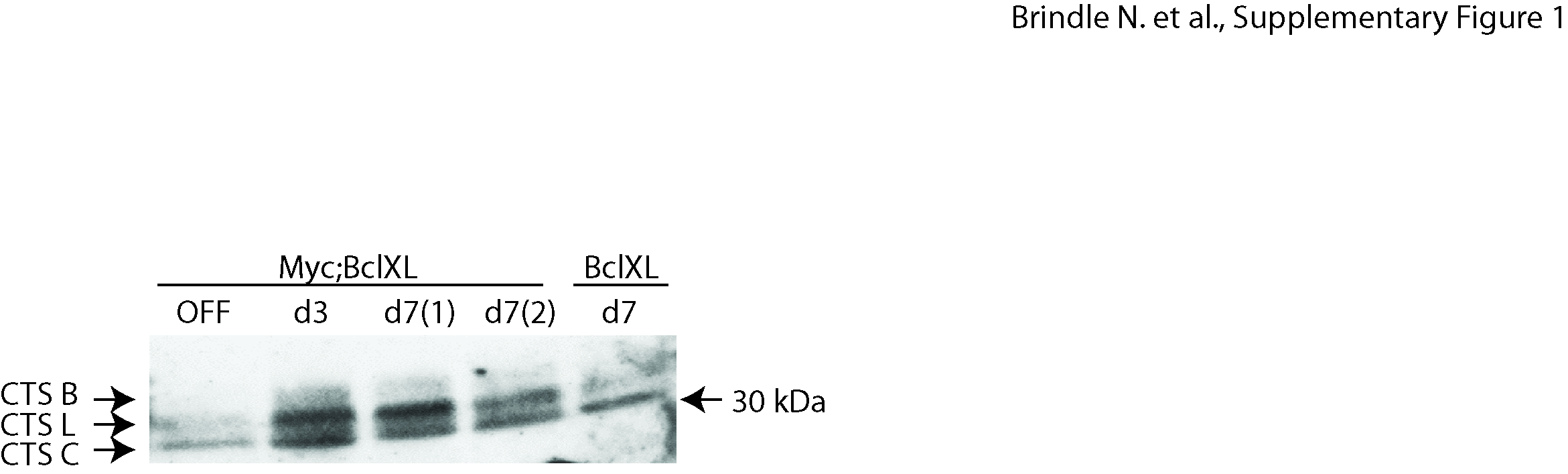

Supplement: S1 Fig — Cathepsin activity profiles for the pancreatic islets from MycER TAM ;Bcl-xL and control Bcl-xL background animals using DCG-04 ABP on tissue lysates. The blot shows activity in untreated islets (OFF), which increases following administration of 4-OHT (Myc-ON) at the timepoints indicated. Two independent islet purifications are presented for MycER TAM ;Bcl-xL animals treated for 7 days. The activity bands corresponding to cysteine cathepsins (CTS) L, B and C are indicated. Molecular weight marker is indicated on the side panel. Note the lack of cathepsin L and cathepsin B activation in samples collected from control Bcl-xL background animals. (TIF) [file pone.0120348.s001.tif]

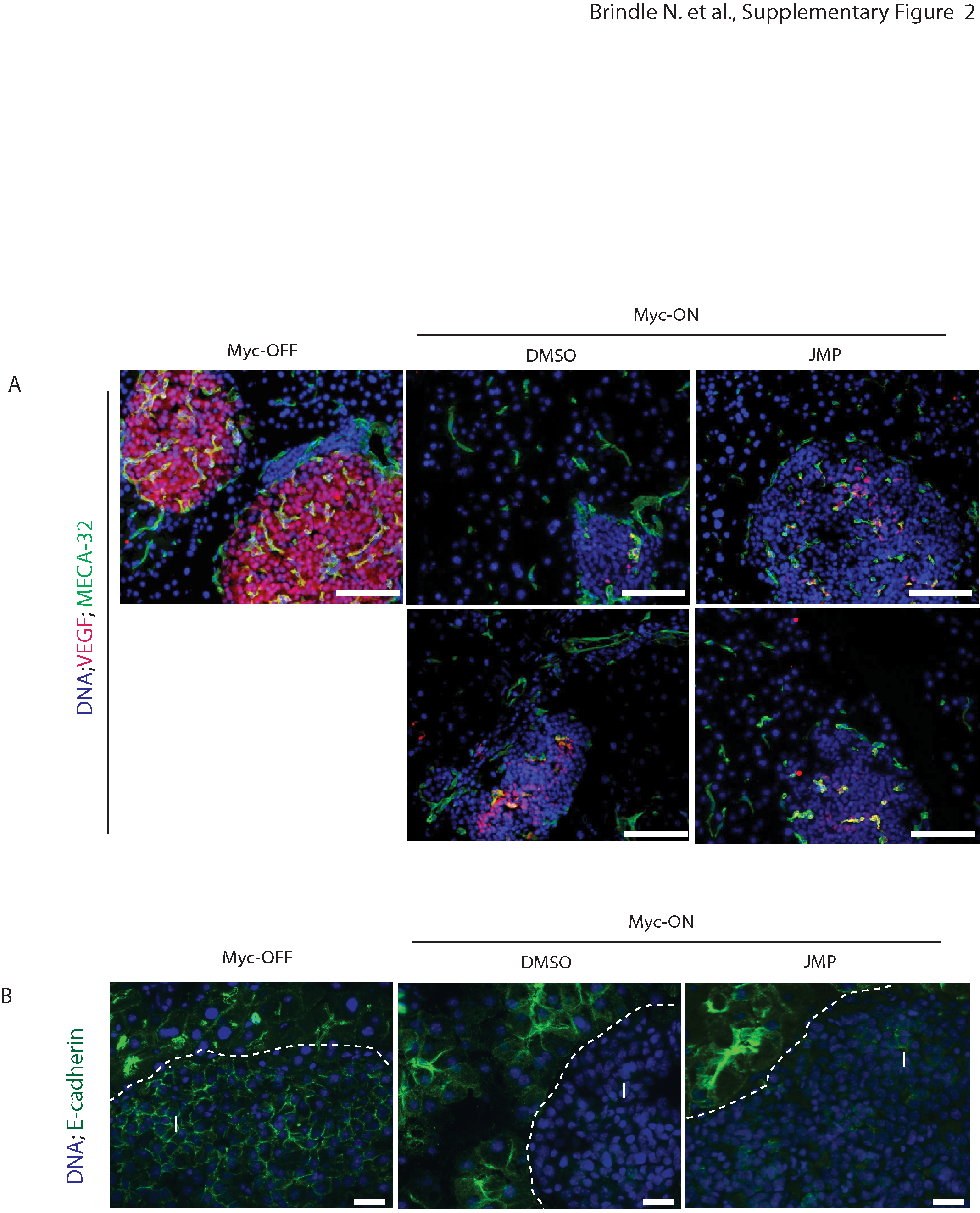

Supplement: S2 Fig — (A) Inhibition of cathepsin activity does not interfere with Myc-induced redistribution of VEGF-A in islets. MycER TAM ;Bcl-x L animals were either untreated or treated daily with TAM for 5 days in conjunction with control vehicle (DMSO) or the broad spectrum cathepsin inhibitor JPM-OEt (JMP). At the times indicated, animals were sacrificed and pancreata isolated, sectioned and stained with anti-VEGF-A antibody (red) and Meca-32 (green). Images collected from two independent animals are presented. The panels are representatives of at least three animals assayed at each data point, immunohistochemical analyses done in duplicate; seven randomized fields per analysis were considered. Scale bars represent 100 μm. (B) Immunohistochemical analysis of E-cadherin in the pancreata collected from the animals described above. The areas positive for E-cadherin expression in inhibitor-treated mice are indicated by arrows. I-islet area is outlined by dotted line. The panels are representatives of at least three animals assayed at each data point, immunohistochemical analyses done in duplicate; five randomized fields per analysis were considered. Scale bars represent 20 μm. (TIF) [file pone.0120348.s002.tif]

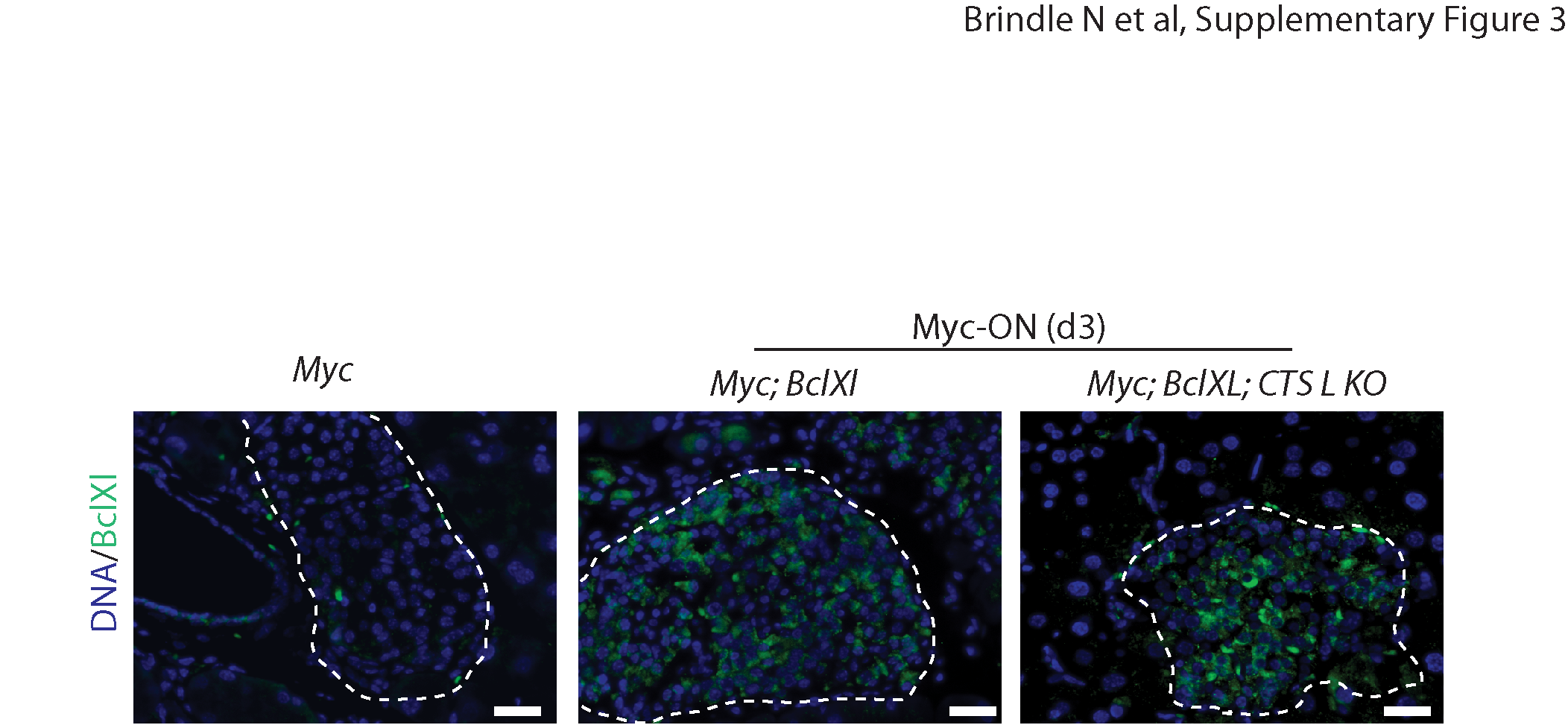

Supplement: S3 Fig — Immunohistochemical analysis of Bcl-xL expression in pancreatic tissues collected from MycER TAM ;Bcl-x L and MycER TAM ;Bcl-x L ;CTSLKO animals subjected to 3-day-treatment with TAM. (Myc-ON, d3). Tissues collected from MycER TAM ;CTSLWT islets were used as a negative control for the staining. Three animals were assayed of each genotype; seven randomized fields per analysis were considered. Scale bars represent 50 μm. (TIF) [file pone.0120348.s003.tif]
